# Association of Adherence to Surfactant Best Practice Uses With Clinical Outcomes Among Neonates in Sweden

**DOI:** 10.1001/jamanetworkopen.2021.7269

**Published:** 2021-05-05

**Authors:** Pontus Challis, Per Nydert, Stellan Håkansson, Mikael Norman

**Affiliations:** 1 Department of Clinical Sciences, Paediatrics, Umeå University, Umeå, Sweden; 2 Division of Pediatrics, Department of Clinical Science, Intervention and Technology, Karolinska Institutet, Stockholm, Sweden; 3 Department of Neonatology, Karolinska University Hospital, Stockholm, Sweden

## Abstract

**Importance:**

While surfactant therapy for respiratory distress syndrome (RDS) in preterm infants has been evaluated in clinical trials, less is known about how surfactant is used outside such a framework.

**Objective:**

To evaluate registered use, off-label use, and omissions of surfactant treatment by gestational age (GA) and associations with outcomes, mainly among very preterm infants (GA <32 weeks).

**Design, Setting, and Participants:**

This population-based cohort study used registry data for 97 377 infants born in Sweden between 2009 and 2018. Infants did not have malformations and were admitted for neonatal care. Data analysis was conducted from June 2019 to June 2020.

**Exposures:**

Timing and number of surfactant administrations, off-label use, and omission of use. Registered use was defined by drug label (1-3 administrations for RDS). Omissions were defined as surfactant not administered despite mechanical ventilation for RDS.

**Main Outcome and Measures:**

In-hospital survival, pneumothorax, intraventricular hemorrhage grade 3 to 4, duration of mechanical ventilation, use of postnatal systemic corticosteroids for lung disease, treatment with supplemental oxygen at 28 days’ postnatal age and at 36 weeks’ postmenstrual age. Odds ratios (ORs) were calculated and adjusted for any prenatal corticosteroid treatment, cesarean delivery, GA, infant sex, Apgar score at 10 minutes, and birth weight *z* score of less than −2.

**Results:**

In total, 7980 surfactant administrations were given to 5209 infants (2233 [42.9%] girls; 2976 [57.1%] boys): 629 (12.1%) born at full term, 691 (13.3%) at 32 to 36 weeks’ GA, 1544 (29.6%) at 28 to 31 weeks’ GA, and 2345 (45.0%) at less than 28 weeks’ GA. Overall, 977 infants (18.8%) received off-label use. In 1364 of 3508 infants (38.9%) with GA of 22 to 31 weeks, the first administration of surfactant was given more than 2 hours after birth, and this was associated with higher odds of pneumothorax (adjusted OR [aOR], 2.59; 95% CI, 1.76-3.83), intraventricular hemorrhage grades 3 to 4 (aOR, 1.71; 95% CI, 1.23-2.39), receipt of postnatal corticosteroids (aOR, 1.57; 95% CI, 1.22-2.03), and longer duration of assisted ventilation (aOR, 1.34; 95% CI, 1.04-1.72) but also higher survival (aOR, 1.45; 95% CI, 1.10-1.91) than among infants treated within 2 hours of birth. In 146 infants (2.8%), the recommended maximum of 3 surfactant administrations was exceeded but without associated improvements in outcome. Omission of surfactant treatment occurred in 203 of 3551 infants (5.7%) who were receiving mechanical ventilation and was associated with lower survival (aOR, 0.49; 95% CI, 0.30-0.82). In full-term infants, 336 (53.4%) of those receiving surfactant had a diagnosis of meconium aspiration syndrome. Surfactant for meconium aspiration was not associated with improved neonatal outcomes.

**Conclusions and Relevance:**

In this study, adherence to best practices and labels for surfactant use in newborn infants varied, with important clinical implications for neonatal outcomes.

## Key Points

**Question:**

What strategies for surfactant treatment of newborn infants are used, and how are these strategies associated with outcomes?

**Findings:**

In this cohort study of 5209 infants who received 7980 surfactant administrations, late surfactant treatment (>2 hours after birth) was provided for 39% of very preterm infants and associated with higher neonatal morbidity. Off-label use occurred in 19% of infants without any association to outcome, and treatment omissions were associated with lower survival.

**Meaning:**

In this study, adherence to best practices for surfactant use in newborn infants varied, with important clinical implications for neonatal outcomes.

## Teaser

This cohort study evaluates registered use, off-label use, and omissions of surfactant treatment by gestational age and associations with outcomes, mainly among very preterm infants (gestational age <32 weeks).

## Introduction

Surfactant therapy for respiratory distress syndrome (RDS) in preterm infants is an advancement in neonatology of the utmost importance.^1,2^ It leads to rapid improvement in oxygenation, decreases the need for ventilator support for RDS, and reduces mortality and air leaks by half.^3,4,5,6^ Early trials also reported a significantly diminished risk of chronic lung disease among survivors.^7^ However, these results have been challenged in more recent studies favoring noninvasive ventilatory support in the delivery room instead of intubation and surfactant administration.^6,8^

As one of the most intensively studied interventions in neonatal care, surfactant therapy rests on solid evidence.^2^ Numerous randomized clinical trials have been performed to determine the efficacy of different surfactant preparations, optimal timing of administration, and optimal dosage.^2,3,4,7,8,9^ Based on this bulk of evidence, recommendations and guidelines have been published,^5,10^ forming the basis for licensed use of surfactant.

While surfactant use within the framework of clinical trials has been described, less is known about surfactant treatment and its outcomes outside such trials. Although observational data on preterm infants receiving surfactant have been published,^11,12,13,14,15,16,17^ these studies provide little information on the number of surfactant administrations and do not cover the full range of gestational ages (GA). In addition, there is often no information on indications for surfactant, the timing of administrations, and compliance to best practices. Off-label use and omission of use are also important areas that have been poorly defined in terms of size of the problem and associations with outcome.^18^

The aim of this study was to assess adherence to surfactant best practices and to test for associations with important clinical outcomes, particularly in very preterm infants (GA <32 weeks). For this purpose, prospectively collected registry data were used to form a national, population-based cohort of nearly 100 000 newborn infants admitted for neonatal care.

## Methods

Ethical approval was obtained from the regional ethics review board in Stockholm, Sweden. A waiver of informed consent was granted because all parents received information regarding the processing of personal data to the Swedish Neonatal Quality Register (SNQ) with a right to opt out and have all personal information removed. This report follows the Strengthening the Reporting of Observational Studies in Epidemiology (STROBE) reporting guideline for cohort studies.^19^

### Participants and Setting

The study was a national, population-based cohort study. The population consisted of all liveborn infants in Sweden between 2009 and 2018 (N = 1 144 432), of whom 67 810 (5.9%) were born preterm, ie, before 37 completed weeks’ GA. The study population consisted of all infants admitted for neonatal care during the same period and reported in the SNQ (n = 107 901). To estimate associations in a more homogeneous population and to reduce potential confounding, newborn infants admitted for inborn errors of metabolism, major malformations as defined by EUROCAT,^20^ chromosomal aberrations, or congenital infections (rubella, cytomegalovirus, toxoplasmosis) were excluded (n = 10 564). The final study population comprised 97 337 infants.

In Sweden, there were 36 neonatal units during the study period. Delivery and initial neonatal care of extremely preterm infants (GA <28 weeks) were centralized to 7 university hospitals in 6 greater regions.

### Data Sources

Open-access statistics provided by Statistics Sweden was used to assess the total number of births.^21^ SNQ has prospectively collected data on neonatal procedures and outcomes for all infants in Sweden admitted to neonatal care. The register has been described and validated, and completeness for preterm infants has been found to be excellent.^22^ Information on extracorporeal membrane oxygenation (ECMO) treatment was validated against the Swedish Intensive Care Register.^23^

### Exposures, Covariates, and Outcomes

Surfactant use was considered the main exposure. In the study period, only 1 porcine surfactant preparation (poractant alfa) was available in Sweden. Licensed use of surfactant included RDS as the indication and administration through an endotracheal tube.^24^ The licensed dose was 100 to 200 mg/kg as a first bolus and 100 mg/kg for subsequent doses. The maximum number of recommended doses was 3.^24^ Use of surfactant outside these recommendations was categorized as off-label use. Omission of use was defined as no surfactant administered despite a diagnosis of RDS and mechanical ventilation starting within 3 days after birth.

SNQ contains information on 3 surfactant variables: surfactant administration (yes or no), the timing of the first surfactant administration (postnatal age in minutes, hours, and days), and the number of administrations. The postnatal age at the first surfactant administration was verified in all cases against the time of birth. Data on timing of the first surfactant administration was missing in 620 of 5209 infants (11.9%), and information on the number of administrations was missing in 257 (4.9%). Rows with missing data were removed to perform analyses on full data sets. The typical practice was surfactant administration via endotracheal tube without immediate extubation. SNQ did not collect information on the use of Less Invasive Surfactant Administration or Intubation-Surfactant-Extubation (INSURE) procedures.

Besides surfactant usage, information was also collected on the following covariates and potential confounders: any prenatal corticosteroid treatment, mode of delivery, GA, postnatal age, sex, Apgar score, birth weight, and *z* score for birth weight (calculation based on the intrauterine growth curve).^25^ In infants with less than 28 weeks’ GA, birth in hospitals without level III neonatal intensive care was included as a covariate.

Diagnoses of lung diseases were categorized as RDS, transient tachypnea of the newborn (TTN), meconium aspiration syndrome (MAS), or other. Diagnostic criteria used were according to the *International Statistical Classification of Diseases and Related Health Problems, Tenth Revision *(*ICD-10*) and a predefined manual for SNQ, available for all users (eTable 1 in the Supplement). If more than 1 diagnosis was reported to the SNQ, all combinations of diagnoses including RDS were coded as RDS, and the combination MAS and TTN was coded as MAS.

Outcomes included in-hospital survival and, in survivors, pneumothorax; intraventricular hemorrhage (IVH) grades 3 to 4, defined according to Papile et al^26^; and indicators of the severity of RDS, such as duration of mechanical ventilation (MV) or continuous positive airway pressure (CPAP) treatment, use of postnatal corticosteroids for lung disease, and supplemental oxygen at 28 days’ postnatal age and at 36 weeks’ postmenstrual age (PMA). For MAS only, outcomes included use of ECMO treatment.

### Statistical Analysis

Data are presented as numbers and percentages or as medians and interquartile ranges (IQRs). For comparisons of surfactant use between regions and comparison between groups, the Pearson χ^2^ test and 2-sample *t* test were used. Changes over time were described with β regression.^27^

To study any associations between surfactant usage (timing, number of administrations, off-label use, omission of use) and outcomes, adjusted odds ratios (aORs) and 95% CIs were calculated using multiple logistic regression. In the logistic regression models, evidence-based (first surfactant administration within 2 hours after birth) and registered uses (number of administrations ≤3 and RDS as indication) were considered reference categories.

In the regression models for very preterm infants, the ORs were adjusted for any prenatal corticosteroids, cesarean delivery, GA in days, sex, Apgar score at 10 minutes, and birth weight *z* score less than −2. Mortality in extremely preterm infants, with less than 28 weeks’ GA, was also adjusted for being born in hospitals without level III neonatal intensive care. In all outcome analyses, infants born at 21 weeks’ GA were excluded (n = 13). The logistic regression model for full-term infants with MAS was adjusted for GA in days, sex, birth weight *z* score less than −2, and Apgar score at 10 minutes.

To estimate goodness of fit in the logistic models, the Hosmer-Lemeshow test was used. *P* < .05 or a 95% CI that does not include 1 for ORs were considered statistically significant differences, and all tests were 2-sided. Statistical analyses were conducted in R version 3.5.2 (R Project for Statistical Computing), and figures were produced using the package ggplot2 version 3.3.3.

## Results

In total, 7980 surfactant administrations were given to 5209 infants (2233 [42.9%] girls; 2976 [57.1%] boys; 4.8% of all admissions for neonatal care): 629 (12.1%) were born at full term; 691 (13.3%) at 32 to 36 weeks; 1544 (29.6%) at 28 to 31 weeks; and 2345 (45.0%) before 28 weeks. The corresponding proportions of admitted infants treated with surfactant were 629 of 58 238 (1.1%), 691 of 30 403 (2.3%), 1544 of 5721 (27.0%), and 2345 of 2975 (78.8%), respectively.

The proportion of extremely preterm infants (GA <28 weeks) treated with surfactant varied from 419 of 580 (72.2%) to 512 of 597 (86.2%) in the 6 greater health care regions of Sweden (*P* = .001). In infants born at 28 to 31 weeks, the proportions treated with surfactant varied between 114 of 552 (20.7%) and 360 of 1231 (29.1%) (*P* = .003). Overall treatment patterns did not change significantly over time (eFigure 1 in the Supplement).

### Diagnoses in Infants Treated With Surfactant

The most common diagnosis in preterm infants treated with surfactant was RDS (4156 of 4580 [90.7%]), followed by TTN (161 [3.5%]). In full-term infants, 336 of 629 receiving surfactant (53.4%) had MAS, 131 (20.8%) had RDS or TTN, and the remaining 162 (25.8%) had other diagnoses (Table 1).

**Table 1.** Respiratory Diagnoses in Infants Treated With Surfactant, Stratified by Gestational Age

Diagnosis	Infants, No. (%)				
	Gestational age, wk				Total (N = 5209)
	<28 (n = 2345)	28-31 (n = 1544)	32-36 (n = 691)	≥37 (n = 629)	
RDS	2219 (94.6)	1392 (90.2)	545 (78.9)	76 (12.1)	4232 (81.2)
TTN	27 (1.2)	72 (4.7)	62 (9.0)	55 (8.7)	216 (4.1)
MAS	0 (0.0)	0 (0.0)	2 (0.3)	336 (53.4)	338 (6.5)
None of the above or missing	99 (4.2)	80 (5.2)	82 (11.9)	162 (25.8)	423 (8.1)

Abbreviations: MAS, meconium aspiration syndrome; RDS, respiratory distress syndrome; TTN, transient tachypnea of the newborn.

### Number of Surfactant Administrations

In total, 7980 surfactant administrations were given: 3082 infants (59.2%) received 1 administration, 1345 (25.8%) received 2, 379 (7.3%) had 3, and 146 (2.8%) had 4 or more administrations. More than half the infants (84 infants [57.5%]) who received 4 or more administrations were born extremely preterm (Table 2; eFigure 2 in the Supplement).

**Table 2.** Infants Treated With 1, 2, 3, or More Surfactant Administrations, by Gestational Age

Gestational age, wk	Infants, No. (%)							
	Surfactant administrations							No surfactant
	1	2	3		≥4		Unknown	
21 (n = 13)	10 (76.9)	2 (15.4)	1 (7.7)	0		0		0
22 (n = 137)	46 (33.6)	49 (35.8)	14 (10.2)	10 (7.3)		1 (0.7)		17 (12.4)
23 (n = 327)	131 (40.1)	98 (30.0)	33 (10.1)	24 (7.3)		20 (6.1)		21 (6.4)
24 (n = 463)	213 (46.0)	142 (30.7)	47 (10.2)	13 (2.8)		14 (3.0)		34 (7.3)
25 (n = 563)	248 (44.0)	141 (25.0)	43 (7.6)	18 (3.2)		19 (3.4)		94 (16.7)
26 (n = 690)	275 (39.9)	133 (19.3)	55 (8.0)	11 (1.6)		15 (2.2)		201 (29.1)
27 (n = 782)	326 (41.7)	113 (14.5)	42 (5.4)	8 (1.0)		30 (3.8)		263 (33.6)
28 (n = 967)	307 (31.7)	103 (10.7)	27 (2.8)	10 (1.0)		17 (1.8)		503 (52.0)
29 (n = 1213)	283 (23.3)	119 (9.8)	22 (1.8)	10 (0.8)		23 (1.9)		756 (62.3)
30 (n = 1520)	241 (15.9)	62 (4.1)	18 (1.2)	4 (0.3)		18 (1.2)		1177 (77.4)
31 (n = 2021)	188 (9.3)	56 (2.8)	17 (0.8)	5 (0.2)		14 (0.7)		1741 (86.1)
32 (n = 3049)	143 (4.7)	51 (1.7)	9 (0.3)	3 (0.1)		12 (0.4)		2831 (92.9)
33 (n = 4634)	103 (2.2)	37 (0.8)	9 (0.2)	4 (0.1)		12 (0.3)		4469 (96.4)
34 (n = 7500)	104 (1.4)	36 (0.5)	6 (0.1)	5 (0.1)		7 (0.1)		7342 (97.9)
35 (n = 8540)	48 (0.6)	32 (0.4)	4 (0.05)	1 (0.01)		6 (0.1)		8449 (98.9)
36 (n = 6680)	35 (0.5)	19 (0.3)	1 (0.01)	1 (0.01)		3 (0.04)		6621 (99.1)
37-39 (n = 29 990)	133 (0.4)	35 (0.1)	5 (0.02)	9 (0.03)		23 (0.1)		29 785 (99.3)
≥40 (n = 28 248)	248 (0.9)	117 (0.4)	26 (0.1)	10 (0.04)		23 (0.1)		27 824 (98.5)

### Timing of First Surfactant Administration and Association With Outcomes

The postnatal age at surfactant administration showed 3 distinctive peaks: the first and largest peak occurred at 10 minutes after birth; the second, 8 to 10 hours’ postnatal age; and the third, at 24 to 30 hours’ postnatal age (eFigure 3 in the Supplement). Timing of the first surfactant administration showed a clear association with GA, with 1513 extremely preterm infants (69.6%) treated with surfactant within 1 hour after birth compared with 90 (15.8%) and 113 (22.6%) in moderately preterm and full-term infants (*P* = .001) (eTable 2 in the Supplement).

In 1364 of 3508 infants (38.9%) with 22 to 31 weeks’ GA, the first surfactant administration was provided after 2 hours’ postnatal age, ie, later than recommended in international guidelines.^10^ This was associated with higher odds of pneumothorax (aOR, 2.59; 95% CI, 1.76-3.83), IVH grades 3 to 4 (aOR, 1.71; 95% CI, 1.23-2.39), use of postnatal corticosteroids (aOR, 1.57; 95% CI, 1.22-2.03), and duration of CPAP (aOR, 1.34; 95% CI, 1.04-1.72) than in those who received the first surfactant administration within 2 hours’ postnatal age. Duration of MV and need of supplemental oxygen at 28 days of postnatal age or at 36 weeks PMA did not show any statistically significant association with timing of first surfactant administration. However, in infants having their first surfactant administration after 2 hours’ postnatal age, in-hospital survival was higher than among those with surfactant administration within 2 hours’ postnatal age (1271 of 1364 [93.2%] vs 1690 of 2144 [78.8%]; aOR, 1.45; 95% CI, 1.10-1.91) (Table 3). The higher survival among infants receiving their first surfactant administration after 2 hours’ postnatal age was confined to infants with 28 to 31 weeks’ GA (aOR, 1.77; 95% CI, 1.03-3.06), whereas there was no statistically significant difference in survival associated with the timing of the first surfactant administration in infants born before 28 weeks’ GA. A sensitivity analysis excluding very preterm infants treated with surfactant for other diagnoses than RDS (278 of 3889 infants [7.1%]) did not alter the results. The distributions of birth characteristics among very preterm infants by surfactant treatment and by timing of the first administration are presented in eTable 3 in the Supplement.

**Table 3.** Surfactant Administration Within or After 2 Hours’ PNA and Associations With Survival, Selected Morbidities, and Additional Respiratory Treatments in Infants With 22 to 31 Weeks’ Gestational Age^a^

Outcome	Infants receiving surfactant, No. (%)		OR (95%CI)	
	≤2 h (n = 2144)^b^	>2 h (n = 1364)	Crude	Adjusted^c^
In-hospital survival	1690 (78.8)	1271 (93.2)	3.67 (2.92-4.67)	1.45 (1.1-1.91)
**Neonatal morbidity in survivors**				
Pneumothorax	60 (3.6)	114 (9.0)	2.68 (1.95-3.71)	2.59 (1.76-3.83)
IVH grade 3-4	148 (8.8)	88 (6.9)	0.78 (0.59-1.02)	1.71 (1.23-2.39)
MV duration >7 d	828 (49.0)	285 (22.4)	0.35 (0.30-0.42)	1.26 (0.98-1.63)
Postnatal corticosteroid use	415 (24.6)	164 (12.9)	0.46 (0.37-0.55)	1.57 (1.22-2.03)
CPAP duration >7 d	1370 (81.8)	820 (65.4)	0.42 (0.35-0.50)	1.34 (1.04-1.72)
Receipt of supplemental oxygen				
28 d PNA	984 (58.2)	509 (40.0)	0.48 (0.41-0.56)	1.08 (0.89-1.32)
36 w PMA	713 (42.2)	333 (26.2)	0.49 (0.42-0.57)	1.07 (0.88-1.32)

Abbreviations: CPAP, continuous positive airway pressure; IVH, intraventricular hemorrhage; MV, mechanical ventilation; OR, odds ratio; PMA, postmenstrual age; PNA, postnatal age.

^a^ Hosmer-Lemeshow test indicated goodness of fit for survival (*P* = .44), pneumothorax (*P* = .40), IVH grades 3 to 4 (*P* = .55), and supplemental oxygen at 36 weeks’ PMA (*P* = .15), whereas goodness of fit was poor (ie, *P* < .05) for the remaining outcomes.

^b^ Reference category.

^c^ Adjusted for prenatal corticosteroid treatment, cesarean delivery, gestational age in days, infant sex, Apgar score at 10 minutes, and birth weight *z* score of less than −2. Mortality was also adjusted for birth in hospitals without level III neonatal intensive care.

### Adherence to Recommendations, Off-Label Use, and Association With Outcomes

The proportion of infants treated with surfactant for the only licensed indication in Sweden, ie, RDS, was 4232 of 5209 (81.2%), meaning that 977 infants (18.8%) received off-label use. The proportion treated with the recommended maximum of 3 surfactant administrations was 4806 of 5209 (92.3%).

Omission of surfactant treatment occurred in 203 of 3551 infants (5.7%) who were receiving mechanical ventilation for RDS. In infants with less than 32 weeks’ GA, treatment omission was associated with lower survival compared with infants who received surfactant treatment (101 of 128 [78.9%] vs 2414 of 2917 [82.8%]; aOR, 0.49; 95% CI, 0.30-0.82). However, proportions of infants treated with postnatal corticosteroids and supplemental oxygen at 28 days’ postnatal age were lower in infants surviving omissions of treatment than in infants treated with surfactant (Table 4).

**Table 4.** Survival, Selected Morbidities, and Treatments in Infants With 22 to 31 Weeks’ Gestational Age Receiving MV for Respiratory Distress Syndrome, Treated With Surfactant and Not Treated With Surfactant^a^

Outcome	Infants, No. (%)		OR (95%CI)	
	Surfactant (n = 2917)^b^	No surfactant (n = 128)	Crude	Adjusted^c^
In-hospital survival	2414 (82.8)	101 (78.9)	0.78 (0.51-1.23)	0.49 (0.30-0.82)
**Neonatal morbidity in survivors**				
Pneumothorax	170 (7.0)	15 (14.9)	2.30 (1.26-3.96)	1.84 (0.98-3.25)
IVH grades 3-4	242 (10.0)	7 (6.9)	0.67 (0.28-1.36)	0.85 (0.35-1.78)
MV duration >7 d	1098 (45.5)	24 (23.8)	0.37 (0.23-0.59)	0.70 (0.36-1.30)
Postnatal corticosteroid use	558 (23.1)	6 (5.9)	0.21 (0.08-0.44)	0.27 (0.09-0.64))
CPAP duration >7 d	1882 (78.7)	60 (60.6)	0.42 (0.28-0.65)	0.78 (0.46-1.33)
Supplemental oxygen				
At 28 d PNA	1346 (55.8)	24 (23.8)	0.25 (0.15-0.39)	0.36 (0.21-0.60)
At 36 w PMA	973 (40.3)	28 (27.7)	0.57 (0.36-0.87)	0.97 (0.58-1.57)

Abbreviations: CPAP, continuous positive airway pressure; IVH, intraventricular hemorrhage; MV, mechanical ventilation; OR, odds ratio; PMA, postmenstrual age; PNA, postnatal age.

^a^ Hosmer-Lemeshow test indicated goodness of fit for survival (*P* = .86), IVH grades 3 to 4 (*P* = .49), and supplemental oxygen at 36 weeks’ PMA (*P* = .18), whereas goodness of fit was poor (ie, *P* < .05) for the remaining outcomes.

^b^ Reference category.

^c^ Adjusted for prenatal corticosteroid treatment, cesarean delivery, gestational age in days, infant sex, Apgar score at 10 minutes, and birth weight *z* score less than −2. Mortality was also adjusted for birth in hospitals without level III neonatal intensive care.

There was no association between 4 or more administrations of surfactant and improved outcomes (eTable 4 in the Supplement). In addition, in full-term infants receiving mechanical ventilation for MAS (336 [55.4%]), there was no observational benefit of surfactant administration (eTable 5 in the Supplement).

## Discussion

In this study, several clinically important observations were made regarding the use of surfactant in Sweden: first, although very preterm infants were the main target group, we found that one-quarter of surfactant administrations were for full-term or moderately preterm infants. Second, significant regional variations in surfactant treatment were found, whereas administration patterns over time were stable. Third, 1 in 5 infants received surfactant off-label regarding indication or number of administrations, without any associations with improved outcome. Fourth, this study demonstrated that adherence to best practice and administering surfactant within 2 hours after birth was associated with lower rates of pneumothorax, IVH grades 3 to 4, and receipt of postnatal corticosteroids in very preterm survivors. However, very early surfactant administration, in this study peaking approximately 10 minutes after birth, was also associated with increased mortality. Finally, omission of surfactant treatment was associated with lower survival in very preterm infants.

We evaluated surfactant use on a population level according to the manufacturer’s label and to the European Consensus Guidelines on the Management of RDS.^10,24^ A European survey study^28^ from 2011 collected data on timing, dose, and preparation from 173 European neonatal intensive care units (NICUs) and concluded that surfactant therapy was implemented according to guidelines.^29^ Our findings of significant off-label use and omissions of treatment challenge this conclusion. Two US network studies from more than 340 NICUs^18,30^ evaluated surfactant use according to the US Food and Drug Administration label, which states that the infant should require ventilatory support for RDS to receive surfactant.^18^ These studies defined INSURE administration and treatment after 30 weeks’ gestation as the 2 most common reasons for off-label use.^18,30^ In addition, they found no significant association between surfactant treatment and lowered mortality or morbidity in preterm infants with GA longer than 30 weeks.^30^

The evidence for treating RDS with surfactant is strong.^10^ For best effect, especially among extremely preterm infants,^3,4^ a policy of early rescue surfactant is advocated.^10,24^ Our findings of significantly lower neonatal morbidity in infants treated with surfactant within 2 hours after birth and of increased mortality in infants in which surfactant therapy was omitted underline the importance of this recommendation. However, and of particular concern, there was lower survival in the group receiving early surfactant administration, also after adjusting for some of the most important factors for infant survival, ie, use of prenatal corticosteroids, GA, Apgar score, and centralization of care.^11,31,32,33^ A possible explanation for an increased mortality is unmeasured confounding by indication, ie, infants with the most severe RDS were treated early but also suffered higher mortality.^34,35^ In addition, we did not control for obstetric complications, such as chorioamnionitis, infections, and bleeding. However, it cannot be excluded that the higher mortality among infants given surfactant very early could be a warning signal. In line with our findings, advanced resuscitation in the delivery room, including endotracheal intubation, particularly of infants in good condition at birth and among less immature infants, has recently been associated with increased mortality.^36^ Lung recruitment, preferably during spontaneous breathing, may also improve the efficacy of surfactant treatment.^37^

The term off-label use refers to a use that is not approved by a national drug agency.^38,39^ Off-label use does not imply that drug use is improper, ineffective, or experimental.^39,40^ In contrast to most drugs used in the NICU,^41,42^ surfactant is extensively studied, with more than 400 publications, providing strong evidence for its use.^2,5,43^ However, TTN was reported as an indication for surfactant therapy in 4% of the infants in this study, most of them born moderately preterm or at full term. The use of surfactant for this self-limiting disorder that requires minimal intervention^44,45^ can be questioned, especially when considering the risk of intubation.^46,47,48,49^ The same concerns are valid for other indications, such as early-onset pneumonia and lung hemorrhage.^50,51^ Surfactant has been shown to decrease oxygen need in congenital pneumonia,^52^ but this recommendation is considered weak and of low evidence.^10^

The natural pool of surfactant has been reported to be as low as 2 to 10 mg/kg in extremely preterm infants compared with approximately 100 mg/kg in the full-term infant.^53,54^ This may explain why some infants, particularly those with very low GA, received 4 or more surfactant administrations. However, it is reasonable to assume that the recommended regimen of 3 administrations resulted in a total dose of 300 to 400 mg/kg, which would be sufficient to substitute surfactant deficiency until the endogenous production begins at 48 to 78 hours’ postnatal age.^10,24^ Our findings of no additional benefit after 3 surfactant administrations are in line with such an interpretation.

Treating MAS with surfactant has been described as potentially being useful.^10,55^ We found no association between surfactant treatment of MAS and a reduction in pneumothorax. This finding is in line with 2 previous systematic reviews^55,56^ but in contrast to a recent multicenter randomized clinical trial showing a reduction of death, ECMO treatment, and pneumothorax in 100 full-term infants treated with surfactant and inhaled nitric oxide for various diagnoses, most commonly MAS and pneumonia.^57^

### Strengths and Limitations

The major strengths of this study are the population-based design of surfactant use in Sweden monitored over a decade, a higher granularity of included variables than in previous studies, and a large number of participants, enabling us to perform adjusted outcome analysis with sufficient power. Although used and analyzed in retrospect, all data were prospectively collected by standardized definitions and procedures.

This study also has limitations. The observational study design limits the conclusions that can be drawn, and no causality of the described associations can be confirmed. The outcome characteristics analysis must be interpreted with caution, as unmeasured or unknown confounding may have occurred. Although the final models for survival were adjusted for several important factors known to affect outcomes, confounding because of the severity of illness may have occurred.^34,35^ Unfortunately, the dosage of surfactant was not available in the register and therefore could not be assessed or analyzed herein.^58^ Two European studies^59,60^ from 2016 and 2019 evaluated the dosage of surfactant and found that both undertreatment and overtreatment were common.

## Conclusions

In this study, there was a high proportion of off-label surfactant use. Early administration of surfactant in RDS as a best practice is supported by our findings of lower incidences of several major neonatal morbidities and of increased mortality if surfactant treatment is omitted. However, the increased mortality associated with immediate surfactant administration in the delivery room, especially in more mature infants, may favor a less invasive approach than that presently practiced in Sweden. Finally, while surfactant treatment might have a role in treating MAS, randomized clinical trials are needed before routine use can be recommended.

## Notes

Supplement. **eTable 1.** Predefined Diagnostic Criteria Used in Swedish Neonatal Quality Register **eTable 2. **Postnatal Age at First Surfactant Administration Stratified by Gestational Age **eTable 3.** Selected Characteristics in Very Preterm Infants (22-31 weeks’ GA) Treated With Surfactant or Not Treated with Surfactant and Surfactant Administration Within of After 2 Hours’ Postnatal Age **eTable 4.** Outcome Characteristics of Very Preterm Infants (22-31 weeks’ GA) Treated With 3 Administrations of Surfactant vs Those With 4 or More Administrations of Surfactant **eTable 5.** Survival, Selected Morbidities, and Treatments in Full-Term Infants Receiving Mechanical Ventilation for Meconium Aspiration Syndrome and Treated Off-Label With Surfactant or Not **eFigure 1. **Proportions of Very (28-31 weeks’ GA) and Extremely Preterm (<28 weeks’ GA) Infants Treated With Surfactant in Sweden by Study Year and Greater Region **eFigure 2. **Proportions of Infants Treated With 1, 2, 3, or More Surfactant Administrations, by GA **eFigure 3. **Density Plot for Postnatal Age at First Administration of Surfactant
