# Supplementary material for: Association of Adherence to Surfactant Best Practice Uses With Clinical Outcomes Among Neonates in Sweden
Source: JAMA Netw Open. 2021 May 5;4(5):e217269. doi: 10.1001/jamanetworkopen.2021.7269 (PMC8100866; doi:10.1001/jamanetworkopen.2021.7269)
Supplement: Supplement. — eTable 1. Predefined Diagnostic Criteria Used in Swedish Neonatal Quality Register eTable 2. Postnatal Age at First Surfactant Administration Stratified by Gestational Age eTable 3. Selected Characteristics in Very Preterm Infants (22-31 weeks’ GA) Treated With Surfactant or Not Treated with Surfactant and Surfactant Administration Within of After 2 Hours’ Postnatal Age eTable 4. Outcome Characteristics of Very Preterm Infants (22-31 weeks’ GA) Treated With 3 Administrations of Surfactant vs Those With 4 or More Administrations of Surfactant eTable 5. Survival, Selected Morbidities, and Treatments in Full-Term Infants Receiving Mechanical Ventilation for Meconium Aspiration Syndrome and Treated Off-Label With Surfactant or Not eFigure 1. Proportions of Very (28-31 weeks’ GA) and Extremely Preterm (<28 weeks’ GA) Infants Treated With Surfactant in Sweden by Study Year and Greater Region eFigure 2. Proportions of Infants Treated With 1, 2, 3, or More Surfactant Administrations, by GA eFigure 3. Density Plot for Postnatal Age at First Administration of Surfactant [file jamanetwopen-e217269-s001.pdf]

Supplemental Online Content

Challis P, Nydert P, Håkansson S, Norman M. Association of adherence to surfactant best practice uses with clinical outcomes among neonates in Sweden. *JAMA Netw Open*. 2021;4(5):e217269. doi:10.1001/jamanetworkopen.2021.7269

eTable 1. Predefined Diagnostic Criteria Used in Swedish Neonatal Quality Register

eTable 2. Postnatal Age at First Surfactant Administration Stratified by Gestational Age

eTable 3. Selected Characteristics in Very Preterm Infants (22-31 weeks' GA) Treated With Surfactant or Not Treated with Surfactant and Surfactant Administration Within of After 2 Hours' Postnatal Age

eTable 4. Outcome Characteristics of Very Preterm Infants (22-31 weeks' GA) Treated With 3 Administrations of Surfactant vs Those With 4 or More Administrations of Surfactant

eTable 5. Survival, Selected Morbidities, and Treatments in Full-Term Infants Receiving Mechanical Ventilation for Meconium Aspiration Syndrome and Treated Off-Label With Surfactant or Not

eFigure 1. Proportions of Very (28-31 weeks' GA) and Extremely Preterm (<28 weeks' GA) Infants Treated With Surfactant in Sweden by Study Year and Greater Region

eFigure 2. Proportions of Infants Treated With 1, 2, 3, or More Surfactant Administrations, by GA

eFigure 3. Density Plot for Postnatal Age at First Administration of Surfactant

This supplemental material has been provided by the authors to give readers additional information about their work.

APPENDIX, supplementary materials on-line.

eTable 1. Predefined Diagnostic Criteria Used in Swedish Neonatal Quality Register

**Diagnosis**	**Diagnostic criteria**
Respiratory distress syndrome (RDS)	Infants ≤28 weeks of GA receiving surfactant within 1 hour after birth<br>OR<br>Arterial pO2 <6.6 kPa or central cyanosis in room air or need of extra oxygen to achieve arterial pO2 ≥6.6 kPa<br>AND<br>chest x-ray compatible with RDS.<br>AND<br>criteria for infection not fulfilled.
Transient tachypnea of the newborn (TTN)	Symptoms of respiratory disorder<br>AND<br>chest x-ray showing some degree of parenchymal changes<br>AND<br>if need of extra oxygen, decrease in FiO_2 within 24 hours<br>AND<br>criteria for infection or other respiratory disorder not fulfilled.
Meconium aspiration syndrome (MAS)	ICD10-code: P24.01

© 2021 Challis P et al. *JAMA Network Open.*

eTable 2. Postnatal Age at First Surfactant Administration Stratified by Gestational Age

	Gestational age, weeks				
**Age 1st surfactant administration, min**	**<28 (N=2345)**	**28-31 (N=1544)**	**32-36 (N=691)**	**≥37 (N=629)**	**Total (N=5209)**
<60	1513 (69.6%)	449 (33.4%)	90 (15.8%)	113 (22.6%)	2165 (47.2%)
<120	110 (5.1%)	82 (6.1%)	26 (4.6%)	50 (10.0%)	268 (5.8%)
<360	289 (13.3%)	324 (24.1%)	109 (19.2%)	105 (21.0%)	827 (18.0%)
<720	94 (4.3%)	163 (12.1%)	87 (15.3%)	94 (18.8%)	438 (9.6%)
>=720	168 (7.7%)	327 (24.3%)	257 (45.2%)	139 (27.7%)	891 (19.4%)
Missing	171	199	122	128	620

Data are numbers and proportions (%) for each GA-strata.

© 2021 Challis P et al. *JAMA Network Open.*

eTable 3. Selected Characteristics in Very Preterm Infants (22-31 weeks' GA) Treated With Surfactant or Not Treated with Surfactant and Surfactant Administration Within of After 2 Hours' Postnatal Age

	**No surfactant, n= 4807 (55.4%)**	**Surfactant, all n=3876 (44.6%)**	**Surfactant, <2hrs n=2144 (61.1%)**
Antenatal corticosteroids, any, No. (%)	3816 (81.3)	3180 (82.8)	1815 (85.4)
Delivery by Cesarean section, No. (%)	2938 (61.4)	2662 (68.8)*	1348 (62.9)
Gestational age, weeks	30.0 (2.0)	27.0 (4.0)*	26.0 (3.0)
Boys, No (%)	2460 (51.2)	2190 (56.5)*	1198 (55.9)
Apgar score at 10 min	10.0 (1.0)	8.0 (2.0)*	8.0 (3.0)
Birth weight, g	1424.0 (519.0)	940.0 (550.0)*	810.0 (447.2)
Birth weight, z-score	-1.0 (1.7)	-0.9 (1.7)	-0.8 (1.6)
Outborn, No (%)	113 (2.4)	407 (10.5)*	251 (11.7)

Data shown as median (IQR) or numbers and proportions (%).
*p<0.001 for group difference compared with no surfactant.

© 2021 Challis P et al. *JAMA Network Open.*

eTable 4. Outcome Characteristics of Very Preterm Infants (22-31 weeks' GA) Treated With 3 Administrations of Surfactant vs Those With 4 or More Administrations of Surfactant

	**3 administrations (N=318)[a]**	**≥4 administrations (N=113)**	**Crude OR (95%CI)**	**aOR[b] (95%CI)**
**In-hospital survival**	224 (70.4%)	70 (61.9%)	0.68 (0.44 - 1.08)	0.75 (0.46 - 1.25)
**Neonatal morbidity in survivors**	n=224	n=70		
Pneumothorax	28 (12.5%)	7 (10.0%)	0.78 (0.3 - 1.78)	0.87 (0.32 - 2.12)
IVH 3-4	41 (18.3%)	17 (24.3%)	1.43 (0.74 - 2.69)	1.53 (0.74 - 3.08)
Duration MV >7days	137 (61.2%)	52 (74.3%)	1.79 (1 - 3.34)	1.89 (0.81 - 4.69)
Postnatal corticosteroids	91 (40.6%)	43 (61.4%)	2.33 (1.35 - 4.07)	2.17 (1.14 - 4.2)
Duration CPAP >7days	192 (86.1%)	62 (88.6%)	1.17 (0.53 - 2.87)	0.99 (0.36 - 2.95)
Supplemental oxygen at 28 days	145 (64.7%)	40 (57.1%)	0.73 (0.42 - 1.26)	0.70 (0.38 - 1.29)
Supplemental oxygen at PMA 36 w	119 (53.1%)	40 (57.1%)	1.18 (0.69 - 2.03)	1.02 (0.55 - 1.88)

[a]Reference category, OR = 1. [b]Adjusted for antenatal corticosteroid treatment, delivery with C-section, GA in days, infant sex, Apgar score at ten minutes and BW-z-score <-2. In addition, mortality was also adjusted for outborn status of the infant.

Data are numbers or proportions (%), or OR with 95% confidence interval (CI). CPAP = continuous positive airway pressure; GA = gestational age; IVH = intraventricular hemorrhage; PMA = Post Menstrual Age; MV = mechanical ventilation; OR = odds ratio; aOR = adjusted odds ratio.

Hosmer-Lemeshow´s test indicated goodness of fit for the outcomes survival (p=0.52), IVH grade 3-4 (p=0.22), Postnatal corticosteroids (p=0.22), Duration CPAP >7days (p=0.45), Supplemental oxygen at 28 days (p=0.07) and Supplemental oxygen at PMA 36 w (p=0.53) whereas goodness of fit was poor (p<0.05) for the remaining two outcomes.

© 2021 Challis P et al. *JAMA Network Open.*

eTable 5. Survival, Selected Morbidities, and Treatments in Full-Term Infants Receiving Mechanical Ventilation for Meconium Aspiration Syndrome and Treated Off-Label With Surfactant or Not

	MAS No Surfactant[a] (N=170)	MAS and Surfactant (N=314)	Crude OR (95%CI)	aOR[b] (95%CI)
**In-hospital survival**	159 (93.5%)	297 (94.6%)	1.21 (0.54 - 2.61)	1.03 (0.43 - 2.38)
**Neonatal morbidity in survivors**	159	297		
Pneumothorax	35 (22.0%)	50 (16.8%)	0.72 (0.44 - 1.17)	0.64 (0.38 - 1.07)
Duration MV >7days	17 (10.7%)	41 (13.8%)	1.34 (0.74 - 2.5)	1.43 (0.77 - 2.76)
ECMO treatment	10 (6.3%)	37 (12.5%)	2.12 (1.06 - 4.62)	1.79 (0.88 - 3.95)

[a]Reference category, OR = 1. [b]Adjusted for GA in days, infant sex, BW-z-score <-2 and Apgar at 10min age

Data are numbers and proportions (%), or OR with 95% confidence interval (CI). GA = gestational age; MV = mechanical ventilation; ECMO = extracorporeal membrane oxygenation; OR = odds ratio; aOR = adjusted odds ratio

© 2021 Challis P et al. *JAMA Network Open.*

eFigure 1. Proportions of Very (28-31 weeks' GA; solid line) and Extremely Preterm (<28 weeks' GA; dashed line) Infants Treated With Surfactant in Sweden by Study Year and Greater Region

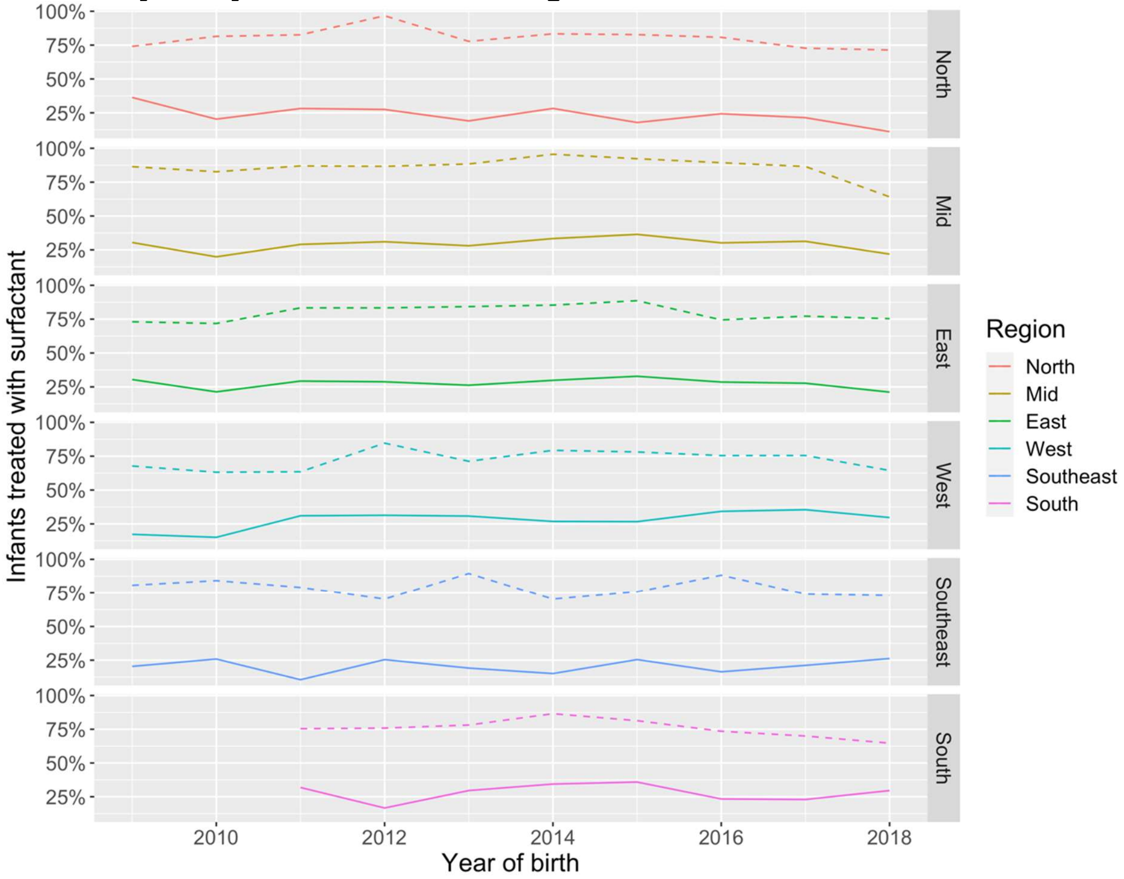

© 2021 Challis P et al. *JAMA Network Open.*

eFigure 2. Proportions of Infants Treated With 1, 2, 3, or More Surfactant Administrations, by GA

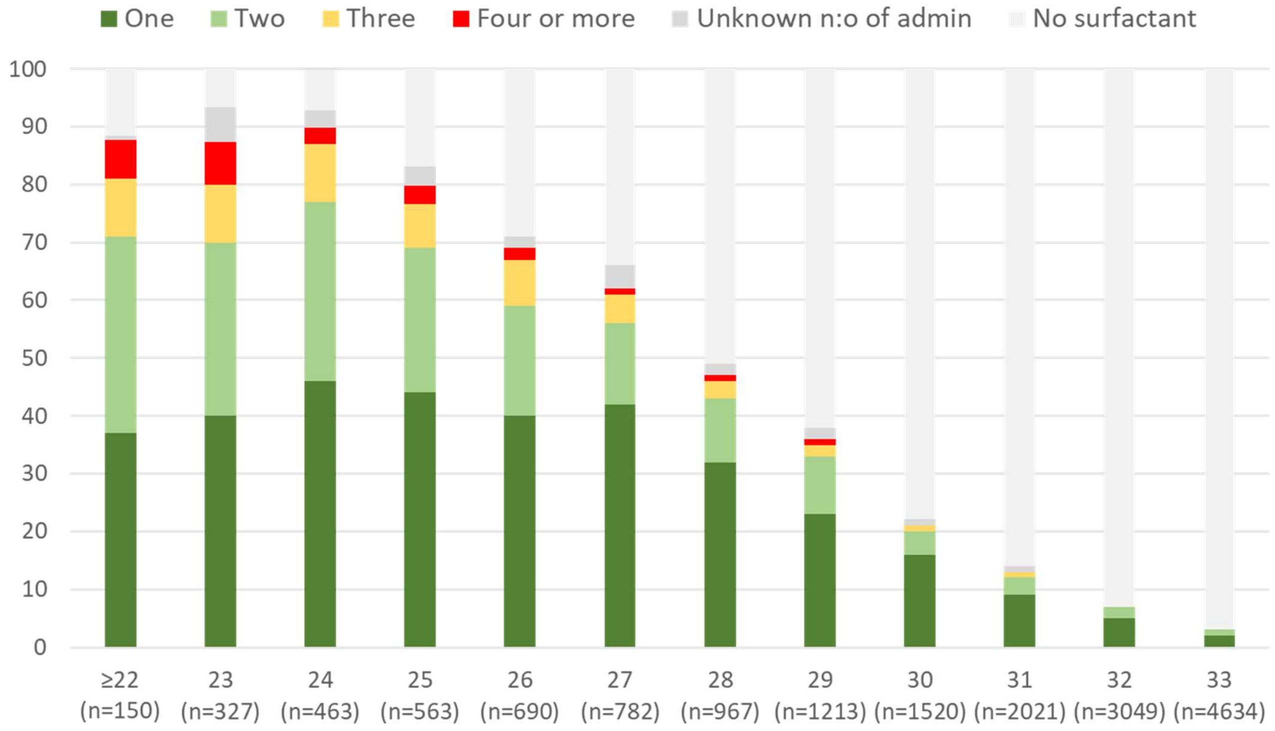

© 2021 Challis P et al. *JAMA Network Open.*

eFigure 3. Density Plot for Postnatal Age at First Administration of Surfactant

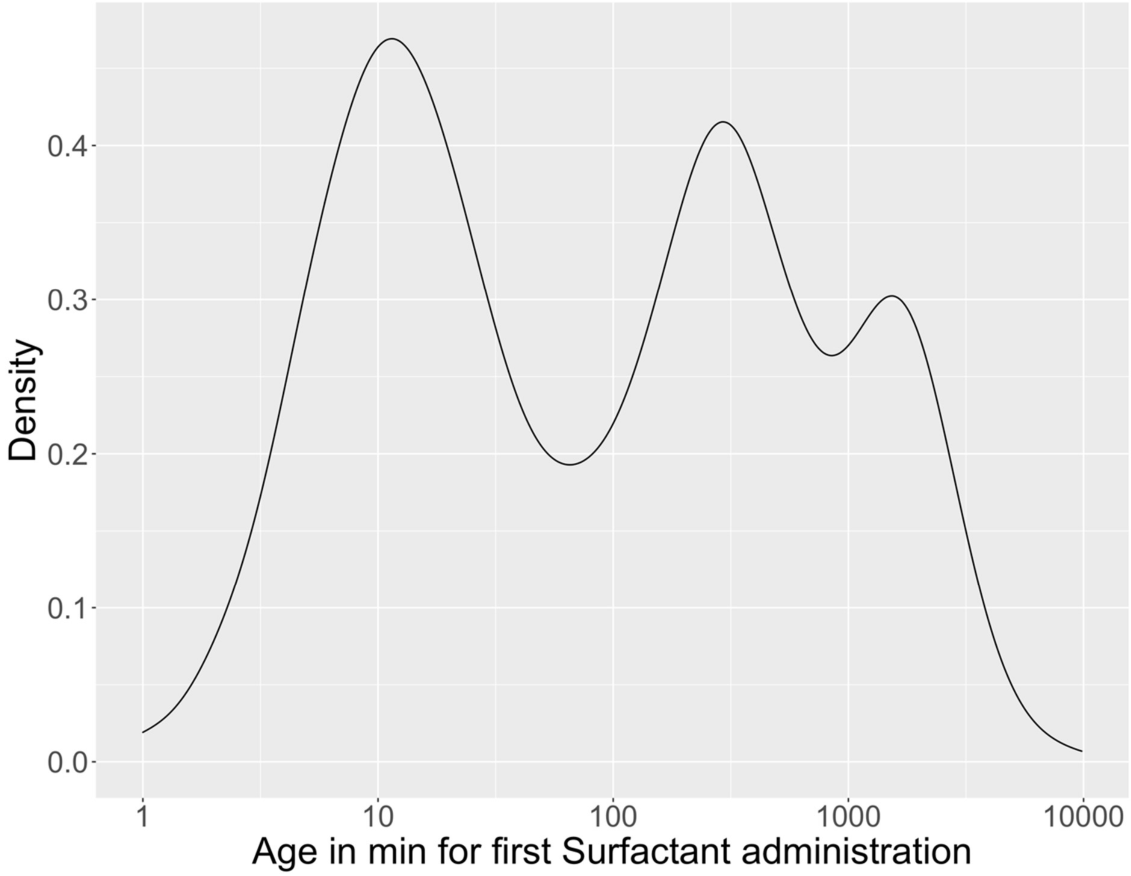

© 2021 Challis P et al. *JAMA Network Open.*
